# Editorial for the Special Issue *Cyclospora cayetanensis* and Cyclosporiasis

**DOI:** 10.3390/microorganisms12020281

**Published:** 2024-01-29

**Authors:** Sonia Almeria, Monica Santin

**Affiliations:** 1 Division of Virulence Assessment, Office of Applied Research and Safety Assessment (OARSA), Center for Food Safety and Nutrition (CFSAN), Food and Drug Administration, Department of Health and Human Services, Laurel, MD 20708, USA; 2 Environmental Microbial and Food Safety Laboratory, Agricultural Research Service, United States Department of Agriculture, Beltsville, MD 20705, USA

Cyclosporiasis is a foodborne diarrheal illness caused by the parasite *Cyclospora cayetanensis*. This parasite has become a major public health and food safety concern as it is responsible for foodborne outbreaks of enteric disease in developed countries, which are mostly associated with the consumption of contaminated fresh produce [1,2,3].

This Special Issue presents important advances, particularly regarding the better detection methods now available, and reviews important aspects of *C. cayetanensis* and cyclosporiasis. It includes two publications about the epidemiology of the parasite in humans, one in Ghana and another in Colombia. The study in Ghana, one of the few studies of this parasite in Africa, confirmed that immunosuppressed patients are more prone and vulnerable to *C. cayetanensis* infection. In endemic countries, the most susceptible populations are children, foreign people, and immunocompromised patients, while in industrialized countries, *C. cayetanensis* affects people of any age [2]. The study in Colombia showed the high endemicity of *C. cayetanensis* in the Colombian Wiwa indigenous people and observed a higher prevalence of *C. cayetanensis* in the rainy season (July–November) compared to a previous study performed in the same area during the dry season (January–April) [4]. *C. cayetanensis *infection is remarkably seasonal, although it varies by geographical region most likely due to human activities, environmental contamination, and the optimal sporulation conditions in each area [2,5].

The development and/or verification of novel detection methods for *C. cayetanensis* for food (two publications) and agricultural water (one publication) are also covered in this Special Issue. The US BAM chapter 19b method performance characteristics and robustness were verified at the Canadian Food Inspection Agency (CFIA) laboratory, for the detection of *C. cayetanensis* using a new detection platform (Bio-Rad CFX96, Bio-Rad, Hercules, CA, USA). The study analyzed imported leafy green, herb, and berry samples, including produce samples under adverse conditions. In a second publication, a new molecular marker based on the mitochondrial genome (cytochrome oxidase gene) was developed for confirmation of the detection of *C. cayetanensis* in food and water samples via conventional PCR (Mit3 PCR) and Sanger sequencing. The method was used to confirm positivity to the parasite in surface water samples that had been found to be positive via dead-end ultrafiltration (DEUF) coupled with quantitative PCR (BAM chapter 19c) [6] at different locations in Maryland, USA [7].

Four publications in the Special Issue reviewed important aspects of *C. cayetanensis*, such as the use of surrogates; life cycle; clinical presentation, pathology, clinical diagnosis, and treatment; and advances in epidemiology and detection methods in the last three years. Surrogates can be used to advance our understanding of *C. cayetanensis* biology, diagnostics, control, and genomics, focusing on opportunities to improve prevention, surveillance, risk assessment, and risk reduction for *C. cayetanensis*. A second publication reviewed advances in the knowledge of the life cycle of *C. cayetanensis*, encompassing a description of the parasitic stages in the human host, including the sexual stages, and a demonstration of the presence of flagella microgametes of *C. cayetanensis* [8,9]. The parasite notably presented small endogenous stages (merozoites and gamonts) in the gallbladder of a man with human immunodeficiency virus and in the small intestine of an immune-competent patient, suggesting that the general life cycle stages are not altered by the immunosuppression of the host [8,9]. The authors would like to note that although these studies have notably advanced the knowledge of the life cycle of *C. cayetanensis*, it will be helpful to have future studies that include cytological smears of intestinal biopsies to definitively establish the dimensions of the endogenous stages of this parasite. Another publication reviewed the clinical presentation, pathology, clinical diagnosis, and treatment of cyclosporiasis cases in detail. Cyclosporiasis is still not often considered by healthcare providers, and many clinical laboratories do not routinely perform testing for this parasite. One manuscript reviewed aspects of the biology, epidemiology, and clinical manifestations of *C. cayetanensis* and provided an in-depth discussion of current laboratory diagnostic methods for clinical cases. On the other hand, a perspective review presented a comprehensive revision of the research published from 2020 to 2023, mainly focusing on epidemiology and detection methods for *C. cayetanensis* in produce and the environment as well as identifying areas in which further research is needed.

As shown in this Special Issue *Cyclospora cayetanensis* and Cyclosporiasis, in the last few years, there have been significant advances in the *Cyclospora* field, such as the expansion of epidemiological worldwide data, the understanding of risk factors, and the development of genotyping assays; however, access to oocysts has been a limiting factor that has considerably hampered what can be learnt about this parasite. Further research will allow for a greater understanding of the genetic diversity of *C. cayetanensis*, its relationship to non-human *Cyclospora* species, and the risk that the presence of *Cyclospora* spp. oocysts poses to our food and water supply [10]. Further research is needed, particularly for trace-back investigations in food and the environment, to provide links between clinical cases and outbreaks, but also to establish measures of parasite viability and to determine the conditions under which sporulation takes place in the environment. The study of the dissemination of oocysts by animals to fields and waters under a One Health approach could also be of interest.
